# Shear Bond Strength of MDP-Containing Self-Adhesive Resin Cement and Y-TZP Ceramics: Effect of Phosphate Monomer-Containing Primers

**DOI:** 10.1155/2015/389234

**Published:** 2015-10-11

**Authors:** Jin-Soo Ahn, Young-Ah Yi, Yoon Lee, Deog-Gyu Seo

**Affiliations:** ^1^Department of Dental Biomaterials Science, Dental Research Institute, School of Dentistry, Seoul National University, 101 Daehak-ro, Jongno-gu, Seoul, Republic of Korea; ^2^Department of Dentistry, Inje University, Seoul Paik Hospital, Marunae-ro 9, Jung-gu, Seoul, Republic of Korea; ^3^Department of Dentistry, Wonju College of Medicine, Yonsei University, 162 Ilsan-dong, Wonju, Republic of Korea; ^4^Department of Conservative Dentistry and Dental Research Institute, School of Dentistry, Seoul National University, Seoul, Republic of Korea

## Abstract

*Purpose*. This study was conducted to evaluate the effects of different phosphate monomer-containing primers on the shear bond strength between yttria-tetragonal zirconia polycrystal (Y-TZP) ceramics and MDP-containing self-adhesive resin cement. *Materials and Methods*. Y-TZP ceramic surfaces were ground flat with #600-grit SiC paper and divided into six groups (*n* = 10). They were treated as follows: untreated (control), Metal/Zirconia Primer, Z-PRIME Plus, air abrasion, Metal/Zirconia Primer with air abrasion, and Z-PRIME Plus with air abrasion. MDP-containing self-adhesive resin cement was applied to the surface-treated Y-TZP specimens. After thermocycling, a shear bond strength test was performed. The surfaces of the Y-TZP specimens were analyzed under a scanning electron microscope. The bond strength values were statistically analyzed using one-way analysis of variance and the Student–Newman–Keuls multiple comparison test (*P* < 0.05). *Results*. The Z-PRIME Plus treatment combined with air abrasion produced the highest bond strength, followed by Z-PRIME Plus application, Metal/Zirconia Primer combined with air abrasion, air abrasion alone, and, lastly, Metal/Zirconia Primer application. The control group yielded the lowest results (*P* < 0.05). *Conclusion*. The application of MDP-containing primer resulted in increased bond strength between Y-TZP ceramics and MDP-containing self-adhesive resin cements.

## 1. Introduction

Yttria-tetragonal zirconia polycrystal (Y-TZP) that is currently used in restorative dentistry contains over 90% zirconium oxide without silica [1]. Y-TZP provides higher fracture toughness and strength compared to other dental ceramics [2]. Unlike other silica-based ceramics, Y-TZP shows a critical weakness in failing to form reliable and durable bonds due to its resistance to hydrofluoric-acid etching [2].

Predictable cementation is one of the most important factors for achieving clinical success with any restorative material, including Y-TZP [3]. However, the cementation method using mechanical and chemical adhesion remains controversial for Y-TZP, unlike for glass or alumina-based ceramics [4]. Previous studies have proven that air abrasion provides micromechanical bonding and that the use of resin cements that consist of 10-methacryloyloxydecyl dihydrogen phosphate (MDP) strengthens the bonds [5–7].

In order to simplify adhesive cementation procedures, self-adhesive resin cements requiring fewer clinical steps have been developed [8, 9]. Various self-adhesive resin cements consist of phosphate monomers, including MDP, and manufacturers suggest that clinicians apply self-adhesive cements to Y-TZP without additional Y-TZP primer [10]. However, studies [6, 9–11] of the bonding efficiency of phosphate monomers in self-adhesive resin cements to Y-TZP have not provided much information on the bonding itself. Thus, it is necessary to evaluate the bond strength between the MDP-containing self-adhesive resin cement and Y-TZP ceramics when each new phosphate monomer-containing primer is used.

Therefore, the aim of this study was to evaluate the effects of different phosphate monomer-containing primers on the shear bond strength between MDP-containing self-adhesive resin cements and Y-TZP ceramics. The null hypothesis was that zirconia primer application would not influence the bonding strength to Y-TZP ceramics.

## 2. Materials and Methods

### 2.1. Specimen Preparation

Ceramic disks of 4 mm thickness, 19 mm diameter, and 100 mm height were obtained by sectioning Y-TZP blocks, which were composed of 97% zirconium dioxide stabilized with a 3% Yttria-Lava Frame (3M ESPE, St. Paul, MN, USA), using a low-concentration diamond blade (Allied High Tech Productions Inc., CA, USA). Under water cooling, the surfaces of each specimen were ground and polished using silicon carbide abrasives of 600-grit. The Y-TZP ceramic specimens were ultrasonically cleaned for 3 min in distilled water prior to sintering as the manufacturer's instructions. The specimens were then embedded in polyethylene molds of 19 mm inner diameter, 21 mm outer diameter, and 12 mm height. For cement bonding, a single side of each disk was left exposed.

### 2.2. Surface Treatments and Bonding Procedure

Depending on the surface treatment method and the resin cement used, 60 specimens were randomly assigned to six groups with 10 specimens per group. The specimens were grouped based on the resin cement used and method of surface treatment. The experimental design and materials used in this study are shown in Tables 1 and 2, respectively. The three groups without air-abrasion treatment were treated with either Metal/Zirconia Primer (Ivoclar Vivadent, Schaan, Liechtenstein) or Z-PRIME Plus (BISCO, Schaumburg, USA) or did not undergo any primer treatment. The three remaining groups were treated with 50 *μ*m grain-sized Al_2_O_3_ particles at a standoff distance of 10 mm and 3.5 bar press for 15 s using air abrasion. The surface was rinsed for 30 s and then air-dried for 30 s after the air abrasion treatment. Either Metal/Zirconia Primer, Z-PRIME Plus treatment, or no primer treatment was performed on the three groups with air abrasion. After being mixed according to the manufacturer's instructions, self-adhesive resin cement (Clearfil SA Luting, Kuraray, Kurashiki, Okayama, Japan) was placed inside a #5 size gel cap (area 16.8 mm^2^). Each specimen with a gel cap was light-cured from all four sides at 600 mW/cm^2^ for 20 s, using an LED curing light unit (Elipar S10, 3M ESPE, St. Paul, MN, USA). At 23 ± 1°C, all specimens were left to polymerize further for 1 h. The specimens were then stored in 37°C distilled water for 23 h. The specimens were subjected to thermocycling (5–55°C for 5000 cycles). The transfer time between baths was 2 s, with a dwelling time of 30 s at each temperature.

### 2.3. Bond Strength Test and Surface Analysis

At a 0.5 mm/min crosshead speed, the adhesive interface of each specimen was loaded with a jig of the universal testing machine (LF-plus, AMETEK Inc., Largo, FL, USA) until failure occurred. The failure modes were observed under a stereomicroscope (45x). The resin bonding on the Y-TZP and fractured surfaces was examined using a scanning electron microscope (SEM; S-4700 FESEM, Hitachi, Tokyo, Japan) at 600x magnification and 10 kV accelerating voltage.

### 2.4. Statistical Analysis

For data analysis, the R programming language (R Foundation for Statistical Computing, Vienna, Austria) [12] was used. The normality of the data and equality of the variance were confirmed. A one-way analysis of variance (ANOVA) and Student–Newman–Keuls multiple comparison test were carried out. The mean difference was considered significant at the level of *P* < 0.05.

## 3. Results

The means and standard deviations for shear bond strength of all groups are presented in Table 3. One-way ANOVA was used to calculate the statistical significance for the different surface treatments (*P* < 0.05). Air abrasion and the use of Z-PRIME Plus were more effective than the control group treatment. The group treated with Z-PRIME Plus after air abrasion showed the best results.

**Figure 1:**
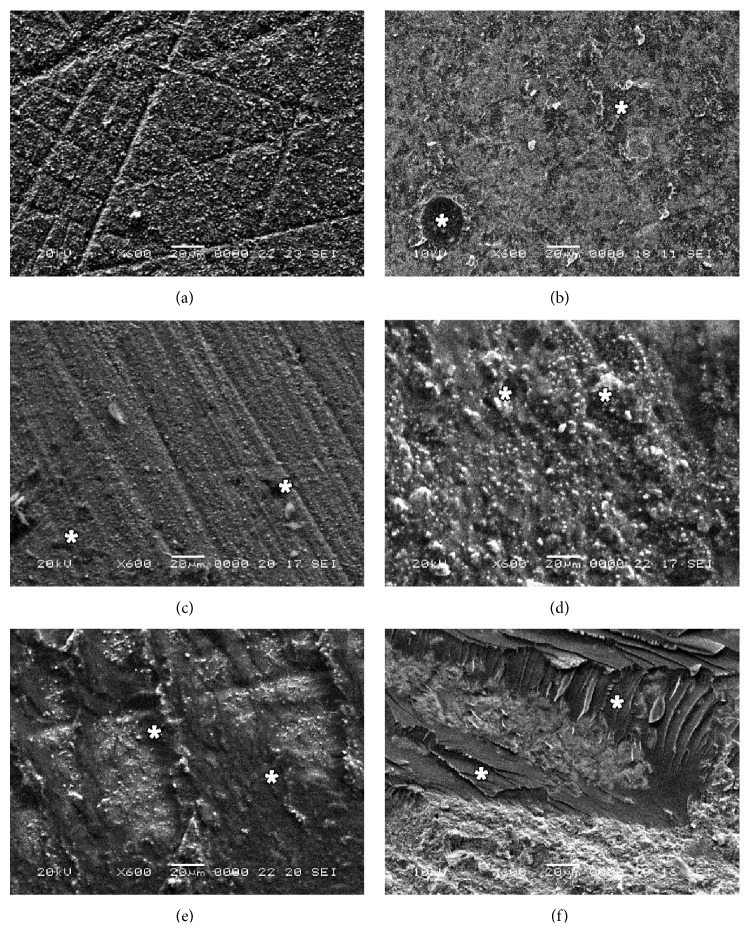
Representative scanning electron microscope images (600x original magnification) of Y-TZP ceramic specimens debonded after cementation with Clearfil SA Luting. (a) Polished Y-TZP; (b) airborne abrasion with 50 *μ*m grain-sized Al_2_O_3_; (c) Metal/Zirconia Primer, a zirconia primer applied on polished Y-TZP; (d) Metal/Zirconia Primer, a zirconia primer applied on Y-TZP after airborne abrasion; (e) Z-PRIME Plus, a zirconia primer applied on polished Y-TZP; and (f) Z-PRIME Plus, a zirconia primer applied on Y-TZP after airborne abrasion. The regions marked with white stars indicate the remaining resin cements.

**Figure 2:**
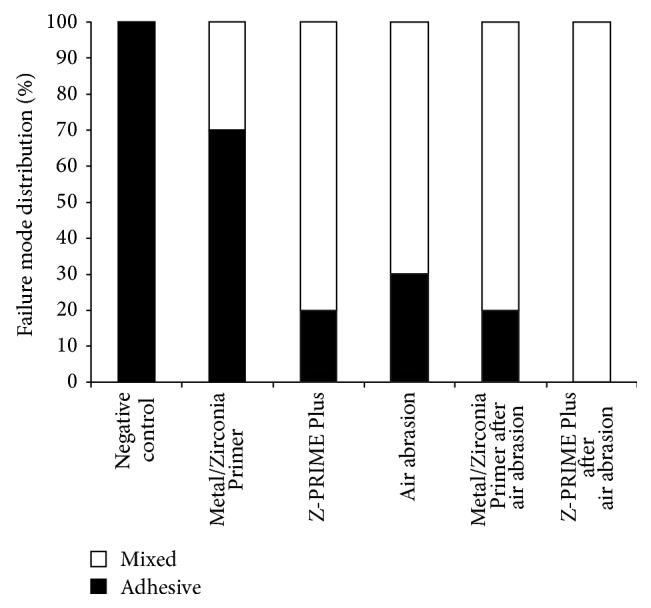
Distribution of failure modes after 10,000 thermocycles.

Figure 1 shows representative SEM images (magnification 600x) for Clearfil SA Luting cement residues on the contact area of the Y-TZP specimens. The failure mode distribution for all samples is shown Figure 2.

## 4. Discussion

This study investigated the effects of different phosphate monomer-containing primers on the shear bond strength between MDP-containing self-adhesive resin cements and Y-TZP ceramics.

The null hypothesis was rejected for the case of MDP as the effective functional monomer in this experiment, and the untreated Y-TZP surfaces showed the lowest bond strengths. High incidence of adhesive failure was observed, leaving the Y-TZP surfaces free of any luting material remnants, which explains the significantly lower bond strength between the self-adhesive resin cement and the untreated Y-TZP surfaces in the control group. This result may be caused by the poor chemical interaction at the interface between the components, at the interface between the MDP component of the Clearfil SA Luting cement and the hydroxyl groups of the Y-TZP ceramics. Other studies [5, 6, 13, 14] have also reported low bond strength when conventional resin cements are used on untreated Y-TZP ceramic surfaces.

Our results indicate that bond strength can be affected by treatment with primer containing MDP, as well as by conventional methods including air abrasion. The bond strength was influenced greatly by 50 *μ*m sized particle air abrasion regardless of the zirconia primer pretreatment. This result is consistent with previous studies [4–6, 15]. The air abrasion method is thought to assist in the progress of resin cement flow into microretentions due to increased roughness and surface energy, which create micromechanical interlocking between the resin cements and Y-TZP [6, 16]. Moreover, air abrasion may generate hydroxyl groups on the Y-TZP surfaces, facilitating the chemical reaction with phosphate monomers [17, 18].

The results of this study show that the MDP containing Z-PRIME Plus promotes durable bonding to Y-TZP. Even though the self-adhesive resin cement included MDP, its functional monomer properties in terms of the amount and flow seemed insufficient to increase the Y-TZP adhesion ability without any pretreatment [6, 19]. Therefore, MDP functional monomers need to be applied to Y-TZP surfaces even if the self-adhesive resin cement contains such monomers. This interpretation is consistent with the results of previous studies [5, 6, 20, 21]. The phosphate ester group of the adhesive monomers and zirconia oxides chemically creates direct bonds [7, 22, 23]. MDP has bifunctional ends that consist of long organic hydrophobic chain molecules. Hydrophilic phosphate ester groups at one end bond strongly to Y-TZP, and vinyl groups react with the monomers of the resin cement at the other end [20, 24].

In the present study, only the MDP-based product, Z-PRIME Plus, had a significantly higher bond strength than did the phosphonic acid-based Metal/Zirconia Primer. The reason is probably that MDP was more effective than phosphonic acid acrylate in Y-TZP surface treatment, though the same phosphate monomer was included in both products [22]. In other studies, MDP-based primers were proven to have higher bond strengths on Y-TZP compared to other primers [7, 21, 22, 25].

It is notable that adhesive failure can be observed in the control group images, whereas the specimens treated with air abrasion, Metal/Zirconia Primer, Z-PRIME Plus, and air abrasion in combination with Z-PRIME Plus presented mixed failures with resin cement. The resin cement remnants can be seen to a relative degree (Figures 1(a)–1(f)).  Figure 1(f) shows a unique ridged appearance, with more resin cement residues in the group with a combination of air abrasion and Z-PRIME Plus application.

The highest bond strength was achieved through the combination of air-abrasion treatment and MDP-based product application. This result may be due to the air-abrasion treatment enhancing the surface wettability and the MDP-containing primer increasing the bond strength, thus improving the chemical affinity. In this group, all specimens indicated mixed fracture patterns, which may be due to the combined effects of the increased contact area with the Y-TZP ceramic surface and the improved chemical interaction with the MDP monomer in Z-PRIME Plus [6, 7]. The combination of MDP-containing primer application and air-abrasion treatment is recommended to achieve strong and durable bonds to Y-TZP using self-adhesive resin cements containing MDP monomers.

## 5. Conclusion

Within the limitations of this study, the following can be concluded.

1. The application of MDP-containing self-adhesive resin cement without pretreatment was not sufficient to improve the bond strength to an untreated Y-TZP surface.
2. MDP-containing primer application seems to be a reliable method for increasing the bond strength between Y-TZP ceramics and MDP-containing self-adhesive resin cements.

## Figures and Tables

**Table 1:** Experimental design for each surface treatment on Y-TZP specimens in this study.

Y-TZP blocks (ground with 600-grit silicon carbide abrasive paper and sintered)					
	↓			↓	
	No air abrasion			Air abrasion	
	↓			↓	

None	Metal/Zirconia Primer	Z-PRIME Plus	None	Metal/Zirconia Primer	Z-PRIME Plus

	↓			↓	

Bonding with Clearfil SA Luting cement (Kuraray, Kurashiki, Okayama, Japan) 5000 thermal cycles between 5 and 55°C, shear bond test (*n* = 10)					

**Table 2:** Characteristics of experimental materials.

Materials	Brand	Product	Manufacturer
Y-TZP	LAVA	97% zirconium dioxide stabilized with 3% Yttria-Lava frame	3M ESPE, St. Paul, MN, USA

Primer	Z-PRIME Plus	HEMA, BPDM, ethanol, and MDP	Bisco Inc., Schaumburg, IL, USA
	Metal/Zirconia Primer	Dimethacrylate, tertiary butyl alcohol, methyl isobutyl ketone, phosphonic acid acrylate, and benzoyl peroxide containing primer	Ivoclar Vivadent, Schaan, Liechtenstein

Resin cement	Clearfil SA Luting	Bis-GMA, TEGDMA, MDP, barium glass, silica, and sodium fluoride	Kuraray, Kurashiki, Okayama, Japan

HEMA: hydroxyethyl methacrylate, BPDM: biphenyl dimethacrylate, MDP: 10-methacryloyloxydecyl dihydrogen phosphate, MPS: 3-methacryloxyprophyltrimethoxy silane, Bis-GMA: bisphenol A-glycidyl methacrylate, and TEGDMA: triethylene glycol dimethacrylate.

**Table 3:** Means and standard deviations of the shear bond strength (MPa) of the samples with Clearfil SA Luting cement and different surface treatments on Y-TZP (*n* = 10).

Priming conditions	Surface conditions	
	No air abrasion (polished)	Air abrasion
None	4.61 (1.13)^A^	9.84 (2.36)^B^
Metal/Zirconia Primer	5.41 (1.66)^A^	10.00 (2.29)^B^
Z-PRIME Plus	10.72 (1.70)^B^	15.23 (1.97)^C^

Different superscripts indicate a statistical difference (*P* < 0.05), and identical superscripts indicate no statistical difference in the designated group after the Student–Newman–Keuls multiple comparison test.
